# Potential Benefits of Second-Generation Human Papillomavirus Vaccines

**DOI:** 10.1371/journal.pone.0048426

**Published:** 2012-11-07

**Authors:** Sorapop Kiatpongsan, Nicole Gastineau Campos, Jane J. Kim

**Affiliations:** 1 Harvard Interfaculty Initiative in Health Policy, Cambridge, Massachusetts, United States of America; 2 Department of Obstetrics and Gynecology, Faculty of Medicine, Chulalongkorn University, Bangkok, Thailand; 3 Department of Health Policy and Management, Center for Health Decision Science, Harvard School of Public Health, Boston, Massachusetts, United States of America; Baylor College of Medicine, United States of America

## Abstract

**Background:**

Current prophylactic vaccines against human papillomavirus (HPV) target two oncogenic types (16 and 18) that contribute to 70% of cervical cancer cases worldwide. Our objective was to quantify the range of additional benefits conferred by second-generation HPV prophylactic vaccines that are expected to expand protection to five additional oncogenic types (31, 33, 45, 52 and 58).

**Methods:**

A microsimulation model of HPV and cervical cancer calibrated to epidemiological data from two countries (Kenya and Uganda) was used to estimate reductions in lifetime risk of cervical cancer from the second-generation HPV vaccines. We explored the independent and joint impact of uncertain factors (i.e., distribution of HPV types, co-infection with multiple HPV types, and unidentifiable HPV types in cancer) and vaccine properties (i.e., cross-protection against non-targeted HPV types), compared against currently-available vaccines.

**Results:**

Assuming complete uptake of the second-generation vaccine, reductions in lifetime cancer risk were 86.3% in Kenya and 91.8% in Uganda, representing an absolute increase in cervical cancer reduction of 26.1% in Kenya and 17.9% in Uganda, compared with complete uptake of current vaccines. The range of added benefits was 19.6% to 29.1% in Kenya and 14.0% to 19.5% in Uganda, depending on assumptions of cancers attributable to multiple HPV infections and unidentifiable HPV types. These effects were blunted in both countries when assuming vaccine cross-protection with both the current and second-generation vaccines.

**Conclusion:**

Second-generation HPV vaccines that protect against additional oncogenic HPV types have the potential to improve cervical cancer prevention. Co-infection with multiple HPV infections and unidentifiable HPV types can influence vaccine effectiveness, but the magnitude of effect may be moderated by vaccine cross-protective effects. These benefits must be weighed against the cost of the vaccines in future analyses.

## Introduction

Prophylactic vaccines against human papillomavirus (HPV), responsible for all cervical cancers and a smaller proportion of other anogenital and oral cancers, represent one of the major breakthroughs in cancer prevention. The two currently available HPV vaccines, which target two of the most oncogenic types, HPV-16 and -18, have the potential to prevent about 70% of cervical cancer cases worldwide; the 4-valent vaccine also targets two non-oncogenic types, HPV-6 and -11, responsible for genital warts. In settings that have not successfully implemented organized cervical cancer screening programs, the introduction of HPV vaccination may provide a critical opportunity for primary prevention of cervical cancer; in settings with organized screening, vaccination could also provide the opportunity to reduce the intensity of screening programs. Indeed, HPV vaccination of young adolescent girls has been shown to be cost-effective in both developed and developing countries [1]–[5].

Despite the great promise for prevention of cervical cancer and other HPV-related cancers, there are several characteristics of current HPV vaccines that may limit their success. Although the 4-valent HPV vaccine has been offered at an unprecedented price ($5 per dose) to the GAVI Alliance, the vaccines remain unaffordable in many parts of the world. Furthermore, the requirement of cold chain in storage and distribution adds another logistical challenge in vaccine accessibility and feasibility. Given regional variation in the proportion of cervical cancers attributable to HPV-16 and -18, the maximum impact of vaccination may be lower than 70%. For example, in Kenya where about 60% of cervical cancers are attributable to HPV-16,-18, reduction in cancer cases could be 10–20% lower than in Uganda, where up to 75% of cervical cancers are attributable to HPV-16,-18.

Second-generation prophylactic HPV vaccines, currently in clinical trials, may hold several advantages over the current vaccines, including protection against additional oncogenic HPV types, less dependence on cold chain storage and distribution, and more efficient delivery mechanisms (e.g., oral and mucosal routes instead of the parenteral route), facilitating integration with other childhood vaccines in routine immunization programs [6], [7]. The second-generation prophylactic HPV vaccine that is most advanced in the pipeline (clinical trial phase III) is a 9-valent (“nonavalent”) vaccine [8]. In addition to the types in the current 4-valent (“quadrivalent”) vaccine (i.e., 6, 11, 16, and 18), the 9-valent vaccine targets five new oncogenic HPV types (i.e., 31, 33, 45, 52, and 58) and has the potential to offer an additional 15–30% reduction in cervical cancer incidence. However, the actual population-level impact will depend on several complicating factors, including: a) the proportion of cancer cases attributable to HPV types targeted by the vaccine, b) the proportion of cancer cases attributable to unidentifiable HPV types, c) the prevalence of co-infections with multiple HPV types, and d) vaccine cross-protective effects against other oncogenic types not covered by the vaccine.

The objective of this study is to provide preliminary estimates of the potential health benefits from the anticipated 9-valent HPV vaccine, exploring the independent and combined effects of these factors. Since reductions in cervical cancer cases are not expected to be observed for several years post-vaccination, disease simulation models that are carefully developed to incorporate information on HPV epidemiology and cervical carcinogenesis can be used to project a range of estimated benefits in the absence of long-term empiric data and under conditions of uncertainty. Used in a decision-analytic framework, such disease simulation models can inform policy makers on how to design new or restructure existing cervical cancer prevention programs in the rapid wake of new technologies [9]–[14].

## Methods

We used an established cervical cancer disease simulation model that had been previously calibrated to two separate countries, Kenya and Uganda, to estimate health benefits in terms of reductions in lifetime risk of cervical cancer from the 9-valent prophylactic HPV vaccine. We selected these two representative countries because of their high cervical cancer incidence rates, the lack of organized cervical cancer screening or HPV vaccination programs, and the different proportions of cervical cancer cases attributable to HPV types 16 and 18. These models had previously been calibrated to fit to epidemiological and clinical data from the respective countries and used to explore the cost-effectiveness of HPV vaccination and cervical cancer screening in five Eastern African countries [1].

We conducted a series of exploratory analyses to estimate both the absolute and incremental benefits of the 9-valent HPV prophylactic vaccine, compared with the benefits from currently-available HPV vaccines (i.e., 2-valent and 4-valent). The influence of several important factors was explored, separately and concurrently:

### I. HPV Type Distribution

The higher the prevalence the vaccine-targeted HPV types are in cervical cancer, the greater the expected reductions in cases from the 9-valent vaccine. Globally, HPV types 16, 18, 31, 33, 45, 52 and 58 are detected in approximately 91.6% (95% CI: 88.5–95.1%) of squamous cell cervical cancers [15], [16]. These seven oncogenic types are associated with 89.4% and 91.4% of cervical cancer cases in Uganda and Kenya, respectively [17]–[20].

While the *total* benefit in cervical cancer prevention from the 9-valent vaccine may not vary much across settings, the *incremental* benefit compared with the benefit of the current vaccines that target only HPV-16 and -18 could vary greatly across populations, depending on the prevalence of these types and the newly-targeted HPV types (i.e., 31, 33, 45, 52 and 58) in cervical cancer. For example, in Kenya, where HPV-16 and -18 contribute to roughly 62.2% of cervical cancer cases, HPV types 31, 33, 45, 52 and 58 are detected in 30.1% of all cases. In contrast, in Uganda, HPV-16 and -18 contribute to 73.9% – and the five other oncogenic types to 17.4% – of cervical cancer cases. Consequently, the added benefits of the 9-valent vaccine over the current vaccines in Kenya could be nearly double the added benefits in Uganda.

**Figure 1:**
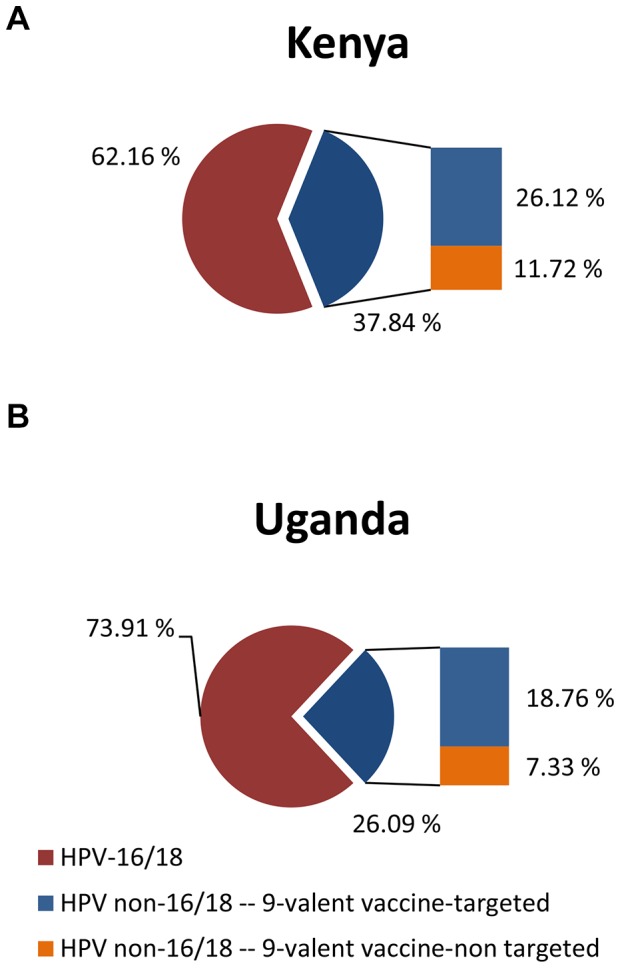
Potential benefits of the 9-valent vaccine by HPV type distribution. The whole pie chart represents all cervical cancer cases in Kenya (1A) and in Uganda (1B). The slice of the blue pie on the right depicts that cases that caused by non-16/18 types. The bar graph adjacent to the blue pie slice shows the proportion of the non-16/18 cases that are the 9-valent vaccine target and are not the 9-valent vaccine target.

To estimate the potential benefit of the 9-valent vaccine in reducing cervical cancer risk, we obtained and used the prevalence of each of the seven oncogenic HPV types targeted by the 9-valent vaccine as the basis to estimate their joint contributions to cancer cases. In Kenya, 62.2% of cervical cancers are caused by HPV-16 and/or -18; [20] the remaining 37.8% of cancer cases are caused by non-16/18 oncogenic HPV types and therefore could possibly be averted by the 9-valent vaccine. We estimated the potential cancer reduction in these 37.8% of cases by calculating the weighted proportions of HPV types 31, 33, 45, 52 and 58 relative to all the non-16/18 oncogenic HPV types. Based on country-specific data on the genotype distribution in cervical cancer cases in Kenya, [18], [20] this proportion is 69.0% and can be interpreted as the contribution of the five newly-targeted oncogenic HPV types in causing cancer among those cases that are not caused by HPV-16 and/or -18. Therefore, solely on the basis of the distribution of HPV types, an additional 69.0% of 37.8% (i.e., 26.1%) of all cervical cancer cases could be reduced from the 9-valent vaccine in Kenya (Figure 1A).

For Uganda, 73.9% of cervical cancer cases are attributable to HPV-16 and/or -18; [19] therefore, the remaining 26.1% of cases are caused by non-16/18 HPV types that may be included in the 9-valent vaccine. Using the proportion of the five newly-targeted HPV types relative to all non-16/18 types in Uganda, [17], [19] an additional 71.9% of those 26.1% (i.e., 18.8%) of cervical cancer cases could be reduced from the 9-valent vaccine in Uganda (Figure 1B).

### II. Co-infection with Multiple HPV Types

When there is more than one HPV type present as the possible cause of cervical cancer, the estimation of vaccine efficacy solely on HPV type distribution could be inaccurate. It is plausible that when more than one HPV type is detected, only one of them is the actual cause of cervical cancer while the other HPV type(s) is(are) just an incidental finding. However, it is also plausible in some cases that there is a required synergistic effect between more than one HPV type in causing cervical cancer. With these diverse biological possibilities, the added benefit of a 9-valent vaccine could be overestimated or underestimated in the cases where multiple HPV types are identified (see Appendix S1 for example scenarios). Based on the empirical data, 5.4% of cervical cancer cases in Kenya and 1.1% of cases in Uganda are associated with more than one of the non-16/18 HPV types [17], [18].

### III. Unidentifiable HPV Types

The proportion of cervical cancer cases that are attributable to HPV infection of unknown genotype could also impact estimates of vaccine benefit. For example, on one extreme, all cases with unidentifiable HPV infections may be caused by one of the oncogenic HPV types that are newly targeted by the 9-valent vaccine, implying that the 9-valent vaccine would yield greater benefits than current vaccines. In contrast, all cases with unidentifiable HPV infections could be caused by HPV types that are not targeted by the 9-valent vaccine or are already targeted by the current vaccines, yielding no additional benefits. Based on empirical data, 3.4% of cervical cancer cases in Kenya and 4.4% of cases in Uganda are associated with HPV of unknown type [17], [18].

**Figure 2:**
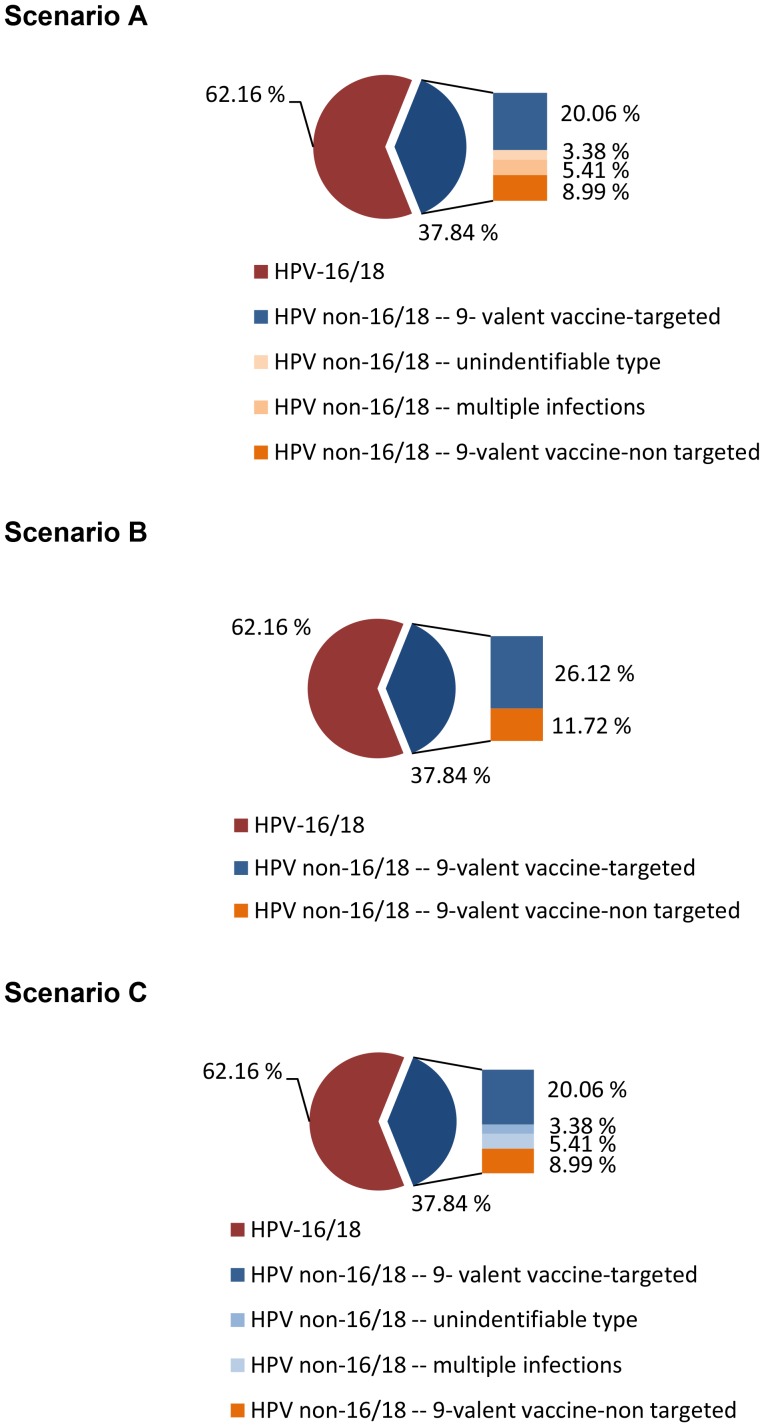
Potential benefits of the 9-valent vaccine by HPV type distribution, multiple HPV infections and unidentifiable types in Kenya. The whole pie chart represents all cervical cancer cases in Kenya. The slice of the blue pie on the right depicts those cases that caused by non-16/18 types. The bar graph adjacent to the blue pie slice shows the proportion of the non-16/18 cases that are associated with multiple infections and unidentifiable types. The remaining non-16/18 cases were estimated to be the 9-valent vaccine target and non-target by their HPV prevalence. In the bar chart adjacent to the pie, blue shading depicts the cases that could be prevented by the 9-valent vaccine. In Scenario A, the 9-valent vaccine could not offer any benefit to prevent cervical cancer with unidentifiable type and multiple infections. In Scenario B, the 9-valent vaccine could prevent some cases with unidentifiable type and multiple infections and the proportion of cases that it can offer benefit was estimated by the prevalence of the five targeted HPV type relative to the prevalence of all non-16/18 types. In Scenario C, the 9-valent vaccine could offer full protective benefits in cases with unidentifiable types and multiple infections.

**Figure 3:**
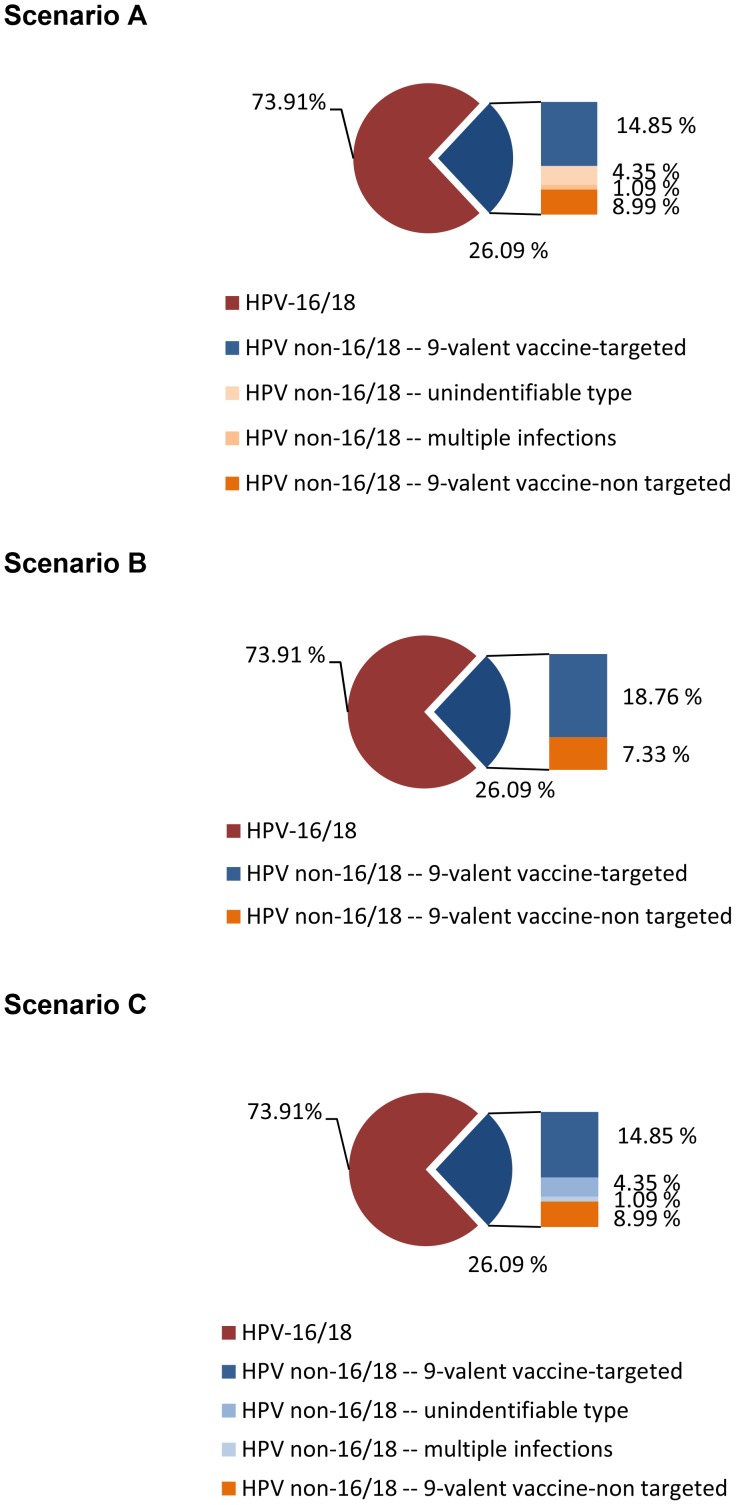
Potential benefits of the 9-valent vaccine by HPV type distribution, multiple HPV infections and unidentifiable types in Uganda. The whole pie chart represents all cervical cancer cases in Uganda. The slice of the blue pie on the right depicts those cases that caused by non-16/18 types. The bar graph adjacent to the blue pie slice shows the proportion of the non-16/18 cases that are associated with multiple infections and unidentifiable types. The remaining non-16/18 cases were estimated to be the 9-valent vaccine target and non-target by their HPV prevalence. In the bar chart adjacent to the pie, blue shading depicts the cases that could be prevented by the 9-valent vaccine. In Scenario A, the 9-valent vaccine could not offer any benefit to prevent cervical cancer with unidentifiable type and multiple infections. In Scenario B, the 9-valent vaccine could prevent some cases with unidentifiable type and multiple infections and the proportion of cases that it can offer benefit was estimated by the prevalence of the five targeted HPV type relative to the prevalence of all non-16/18 types. In Scenario C, the 9-valent vaccine could offer full protective benefits in cases with unidentifiable types and multiple infections.

Based on these three sources of uncertainty, we constructed and analyzed three extreme scenarios in our study, assuming among cervical cancer cases that: (A) there is no overlap between unidentifiable HPV types and multiple infections with HPV types that are targeted by the 9-valent vaccine, and therefore, the 9-valent vaccine does not offer any benefit in these cases (Figure 2A for Kenya and Figure 3A for Uganda), (B) there is some overlap between unidentifiable HPV types and multiple infections with vaccine-targeted HPV types, and therefore, the 9-valent vaccine has the ability to prevent some cases with unidentifiable types and multiple infections, defined as a function of the prevalence of the five targeted HPV types relative to the prevalence of all non-16/18 types (Figure 2B for Kenya and Figure 3B for Uganda), and (C) there is complete overlap between unidentifiable HPV types and multiple infections with vaccine-targeted HPV types and therefore the 9-valent vaccine offers full protective benefits in cases with an unidentifiable type and multiple infections (Figure 2C for Kenya and Figure 3C for Uganda).

### IV. Vaccine Cross-protection

Clinical trials have demonstrated that current HPV vaccines exhibit cross-protective effects against non-16/18 oncogenic types, [21]–[23] including the five oncogenic types that are included in the 9-valent vaccine, in which case the added benefit of the 9-valent vaccine over the current vaccines may be diminished. When unadjusted for co-infection with multiple HPV types, this cross-protective effect associated with the bivalent vaccine was estimated in clinical trials to be roughly 53.0% in preventing precancerous lesions caused by HPV types 31, 33, 45, 52, or 58, and 61.9% for lesions caused by any oncogenic HPV type [21]–[23]. After adjustment for co-infections between (a) HPV-16 or -18 and (b) other non-16,-18 oncogenic types, the estimate of cross-protection against lesions decreased to 37.4% [21]. It is also possible that the 9-valent vaccine itself will exhibit cross-protective effects against other non vaccine-targeted HPV oncogenic types in a similar manner as the current vaccines.

We incorporated cross-protective effects against other non-vaccine types into our existing Scenarios A, B and C with four possible levels of cross-protection: (1) none (0% cross-protection), (2) low (7.4% cross-protection), (3) moderate (37.4% cross-protection), and (4) high (58.2% cross-protection) [21]. By varying all sources of uncertainty simultaneously, the simulation model can determine a full range of potential benefits from the 9-valent vaccine. Overall population *absolute benefit* would be expected to be lowest in a scenario where none of the cases with multiple HPV infections and unidentifiable HPV type were caused by HPV infections targeted by the 9-valent vaccine. The most favorable scenario for the 9-valent vaccine would be if all cancer cases with multiple HPV infections and unidentifiable HPV types were attributable to at least one of the newly-targeted HPV types and were fully protected by the 9-valent vaccine.

### Model Inputs and Assumptions

Several assumptions were required for the analysis: (a) girls receive the vaccine (either the 9-valent HPV vaccine or the current HPV-16,-18 vaccines) by age 12 prior to sexual debut; and (b) all vaccine recipients complete the full three-dose series (100% vaccine compliance rate) that is required for current vaccines and was assumed for the 9-valent vaccine. The primary outcomes were absolute and relative reductions in lifetime risk of cervical cancer, compared to no intervention.

## Results

**Figure 4:**
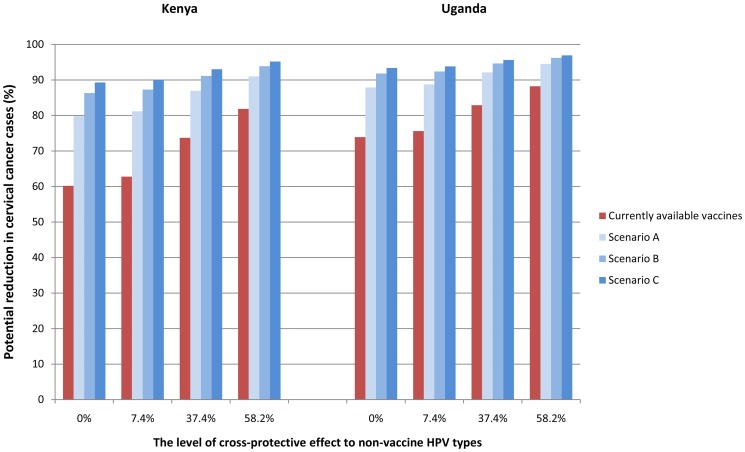
Potential benefits of the 9-valent vaccine by HPV type distribution, multiple HPV infections, unidentifiable types, and vaccine cross-protective effects in Kenya and Uganda. The bar graph depicts the potential benefits in reduction (%) of cervical cancer cases of currently available vaccines and the 9-valent vaccine under the Scenarios A (no benefit to cases with unidentifiable HPV types and HPV co-infections), B (partial benefit to cases with unidentifiable HPV types and HPV co-infections), and C (full benefit to cases with unidentifiable HPV types and HPV co-infections) with varying levels of vaccine-cross protective benefits in Kenya and Uganda.

Figure 4 shows the absolute reductions in lifetime risk of cervical cancer by the currently available HPV-16/18 vaccines and by the 9-valent HPV vaccine under scenarios A, B, and C across the four different assumptions of vaccine cross-protection against non-vaccine HPV types.

For the current vaccines, the absolute reductions in lifetime risk of cervical cancer were 60.2% in Kenya and 73.9% in Uganda. Assuming vaccine cross-protection against non-vaccine types, these reductions increased to 81.9% in Kenya and 88.2% in Uganda.

In Scenario A, the 9-valent vaccine yielded absolute risk reductions of approximately 79.8% in Kenya and 87.9% in Uganda, when there was no cross-protection against non-vaccine types. The reductions were as high as 91.0% in Kenya and 94.5% in Uganda, under assumptions of high cross-protection against non-vaccine types.

In Scenario B, the 9-valent vaccine increased absolute risk reductions to approximately 86.3% in Kenya and 91.8% in Uganda, without cross-protection against non-vaccine types, and were as high as 93.9% in Kenya and 96.2% in Uganda, with high levels of cross-protection against non-vaccine types.

In Scenario C, the reductions in lifetime risk of cervical cancer associated with the 9-valent vaccine were greatest, ranging from 89.3% to 95.2% in Kenya and 93.4% to 96.9% in Uganda, depending on assumptions of cross-protection.

## Discussion

Across varied scenarios of uncertainty, our simulation model projects that the 9-valent vaccine could reduce lifetime risk of cervical cancer by 79.8% to 95.2% in Kenya and 87.9% to 96.9% in Uganda. In comparison, currently-available vaccines could offer protection against 60.2% to 81.9% of cervical cancers in Kenya and 73.9% to 88.2% of cervical cancers in Uganda. The added benefits of the 9-valent vaccine over current vaccines therefore ranged from 9.2% to 29.1% in Kenya and from 6.3% to 19.5% in Uganda.

Our study provides evidence that uncertainties surrounding HPV type distribution in cancer, unidentifiable HPV types, co-infection with multiple HPV infections, and cross-protection against non-vaccine types can lead to variable estimates of both the absolute and incremental benefits of the 9-valent vaccine. The added benefits were most favorable when the 9-valent vaccine could fully protect against unidentifiable HPV types and multiple infections (Scenario C) with an assumption that none of the vaccines provide cross-protection against non-vaccine HPV types. The added benefits were least favorable when cases with unidentifiable types and multiple infections were not attributable to types targeted by the 9-valent vaccine (Scenario A) with an assumption that all the vaccines provide high cross-protection against non-vaccine types.

As expected, HPV type distribution in cancer had a greater impact on the added benefits of the 9-valent vaccine (compared to current vaccines) than the absolute benefits. Results showed that the added benefit of the 9-valent vaccine in Kenya, where cervical cancer is less attributable to HPV-16 and -18, was approximately 1.5 times greater than in Uganda, where cervical cancer is more commonly caused by HPV-16 and/or -18. The greater magnitude in added benefits in Kenya persisted in all scenarios from no cross-protection to a high level of cross-protection and from no benefit (Scenario A) to full benefit (Scenario C) against unidentifiable types and multiple infections.

This finding suggests that in a geographic area where cervical cancer is less attributable to HPV-16 and -18, the 9-valent vaccine could offer greater added benefits, compared to the current vaccines. Based on epidemiological data of HPV type distribution in cancer from the WHO/ICO Information Centre on Human Papilloma Virus (HPV) and Cervical Cancer (available at http://www.who.int/hpvcentre/en/), roughly 68.0%, 68.5%, 70.3%, 73.0% and 75.1% of cervical cancer cases were associated with HPV-16 and -18 in Asia, Africa, the Americas, Europe and Oceania continents, respectively [24]. Therefore, based on the type distribution of HPV -16 and -18 in cervical cancer alone, populations in Asia could possibly gain relatively higher added benefits from the 9-valent vaccine than other populations. Based on the reported prevalence of the five additional HPV types in cervical cancer targeted by the 9-valent vaccine, up to 26.6%, 20.5%, 18.0%, 14.2% and 3.1% of cervical cancer cases in Asia, Africa, Americas, Europe and Oceania continents could be prevented by the 9-valent vaccine [24]. As showcased in our analysis, in addition to the causal attribution of HPV genotypes, other important factors such as the prevalence of multiple infections, prevalence of unidentifiable types, vaccine cross-protective properties, and the availability of a cervical cancer screening program in a particular population must be taken into consideration. Distributive patterns of HPV types in cervical cancer can vary greatly across countries within the same continent as was shown in Kenya and Uganda. As a result, specific epidemiological data in the population of interest should be used when available to estimate the context-specific benefits of the 9-valent vaccine.

Our results also suggest that uncertainty from unidentifiable HPV types and co-infection with multiple HPV types could affect the lifetime risk reductions in cervical cancer by approximately 4.2–9.5% in Kenya and approximately 2.4–5.5% in Uganda, based on the difference between the two extreme scenarios (Scenarios A and C) with the varying degrees of cross-protection against non-vaccine types. The impact of the uncertainty from unidentifiable types and co-infection with multiple types decreased as the level of cross-protection against non-vaccine types increased.

Cross-protection against non-vaccine types was expected to reduce the added benefits of the 9-valent vaccine over the benefits from the current vaccines. Our results demonstrated that, with moderate cross-protection against non-vaccine types, the added benefits from the 9-valent vaccine could decrease by 6.4–9.8% in Kenya and by 4.8–6.7% in Uganda, compared to no cross-protection against non-vaccine types. With high cross-protection against non-vaccine types, the added benefits from the 9-valent vaccine were reduced by 10.5–15.8% in Kenya and by 7.7–10.8% in Uganda, in comparison to a scenario with no cross-protection.

The findings from our exploratory analysis underscore that estimates of the incremental benefits from the 9-valent HPV vaccine compared to the current HPV-16/18 vaccines may vary widely subject to several uncertainties, ranging from cancer reductions of 9.2% to 29.1% in Kenya and 6.3% to 19.5% in Uganda. Despite the utility of our disease simulation model in projecting preliminary estimates of population-level vaccine benefits under complex interactions of various uncertainties, our analysis has several limitations. First of all, we did not model the infection from each of the HPV types individually. We classified all non-16, non-18 HPV types into either the group of five additional types targeted by the 9-valent vaccine or the group of the remainder types. We then made a simplifying assumption that the contributions to cervical cancers from all of these non-16, non-18 types were directly proportionate to their prevalence in non-16, non-18 cancer cases. This estimation may be imprecise if some oncogenic HPV types are more likely than others to be incidental infections, but in the absence of such information, we only modeled the contributions to cervical cancer based on prevalence.

Secondly, we did not vary the levels of vaccine uptake and adherence to the full three-dose vaccine series. Perfect vaccine uptake is unlikely even in developed countries. Indeed, a recent national survey in the United States showed that fewer than 1 out of 3 teenage girls between 11 and 17 years old had received at least one dose of currently available HPV vaccines [25]. However, by assuming complete coverage, we were able to estimate the maximum potential reductions of cervical cancer under the different scenarios. In a real-world situation where uptake is imperfect, the potential benefit of the 9-valent vaccine is expected to decrease roughly proportionately to the percentage of uptake. For example, in Kenya, if complete uptake offers 79.8% reduction in lifetime risk of cervical cancer, then 50% uptake will offer approximately half, or 40%, reduction. Note that this estimate does not take into consideration potential herd immunity benefits that may come into play when vaccine uptake is incomplete and may make the reduction even greater.

To date, there is no evidence suggesting that the 9-valent HPV vaccine will result in differentially higher uptake than currently available vaccines. While protection against more HPV types could be a strong incentive for individuals to obtain the vaccine, there is not yet a clear advantage in how the vaccine will be administered (e.g., parenteral versus oral routes) or in the number of doses required. Vaccine uptake will continue to be a concern for the 9-valent vaccine as well as for currently available vaccines.

In this study, we did not examine the impact of screening programs on the absolute and added benefits from the 9-valent vaccine; therefore, generalizability to settings that have screening programs may be limited. In settings where effective screening programs exist, we expect that the absolute and added health benefits from the 9-valent vaccine would be reduced. Generally, with more effective screening programs, the benefits of vaccination are expected to be lower. This trend applies to both currently available vaccines and the 9-valent vaccine. Countries without an effective cervical cancer screening program are likely to achieve greater added benefits from the 9-valent vaccine, compared to countries where effective screening programs are in place.

We did not explore the benefits of vaccinating both boys and girls with the 9-valent vaccine relative to vaccinating both sexes with currently available vaccines. We expect that vaccination of both sexes with the 9-valent vaccine would further reduce lifetime risk of cervical cancer. Male HPV vaccination would also result in direct health benefits to males (by preventing male HPV-related diseases, such as genital warts, and anal, penile, and oral cancers), as well as contribute to reduced HPV transmission in the population, thus providing “indirect” benefits to sexual partners. However, the burden of HPV-related diseases in males is relatively low, and we have learned from previous analyses that the added benefits from including boys in the vaccination program would likely be less than the added benefits from increasing uptake in girls [26]. Furthermore, there is limited information on the prevalence and type distribution of the five additional HPV genotypes in males and the transmission of the five additional HPV types between sexual partners, which are critical inputs to the evaluation. As more data become available on HPV in males, an evaluation of the benefits of vaccination of both sexes with the 9-valent vaccine will be a priority. Ultimately, the decision to expand the target population for HPV vaccination beyond young girls will need to include considerations of program costs, the burden of HPV-related diseases in males, the feasibility of achieving effective coverage rates, and the desirability to implement a gender-neutral vaccination program for ethical, cultural, social, or practical reasons.

There are other noteworthy limitations. The estimated benefits from HPV vaccination were restricted to squamous cell carcinomas of the cervix, as there is limited clinical and epidemiological information on non-squamous cell types of cervical cancer. As a result, the estimates may not be applicable to prevention of non-squamous cell cancers. In addition, we did not make any assumptions regarding the correlations between the four sources of uncertainty in estimating vaccine benefits. For example, we did not assume that cancer cases with unidentifiable types and multiple infections occurred with a positive correlation. Information from clinical trials may reveal negative or positive correlations among these factors, and modeling the interactions between different uncertainties may be feasible with such data.

Other factors should be considered in policy decisions regarding adoption of the 9-valent vaccine. In this analysis, we assumed that neither the current vaccines nor the 9-valent vaccine have serious adverse side effects and compared only vaccine benefits in terms of cancer reduction. Costs of the vaccines and implementation are important factors that should be incorporated in future studies to assess the value of the added benefits of the 9-valent vaccine.

### Conclusions

The 9-valent HPV prophylactic vaccine is expected to offer greater protection against cervical cancer than current vaccines. The magnitude of this added benefit, however, is uncertain due to the complex interactions of several factors; important among them are HPV type distribution in cancer cases, cases that are attributable to co-infections with multiple HPV types or unidentifiable type(s), and vaccine cross-protection against non-vaccine types. In this study, we leveraged a disease simulation model of HPV-induced cervical cancer to estimate health gains in terms of cervical cancer reductions associated with the 9-valent vaccine under different scenarios. Type distribution of HPV in cancer cases, and cancers attributable to multiple HPV infections and unidentifiable HPV types can influence vaccine effectiveness individually and jointly, but the magnitude of influence may be moderated by vaccine cross-protective effects. These benefits must be weighed against the cost of the vaccines in future analyses.

### Ethics Committee Approval

Ethics committee approval was not required for this study.

## Supporting Information

Appendix S1 **Multiple HPV infections and potential vaccine benefits.** This supplemental appendix gives examples of scenarios to show how co-infection with multiple HPV types can influence the estimation of vaccine benefits of the 9-valent HPV vaccine.
(DOC)
