# Supplementary material for: Potential Benefits of Second-Generation Human Papillomavirus Vaccines
Source: PLoS One. 2012 Nov 7;7(11):e48426. doi: 10.1371/journal.pone.0048426 (PMC3492348; doi:10.1371/journal.pone.0048426)
Supplement: Appendix S1 — Multiple HPV infections and potential vaccine benefits. This supplemental appendix gives examples of scenarios to show how co-infection with multiple HPV types can influence the estimation of vaccine benefits of the 9-valent HPV vaccine. (DOC) [file pone.0048426.s001.doc]

**Supplemental Appendix S1**

Accompanying “Potential Benefits of Second-generation Human Papillomavirus Vaccines”

**Multiple HPV infections and potential vaccine benefits**

Below are example scenarios on how multiple HPV infections can influence the estimation of vaccine benefits of the 9-valent HPV vaccine.

**Scenario X**: A cervical cancer case with the presence of both HPV types 18 and 33

If HPV type 18 is the only causative agent for cervical cancer and the presence of HPV type 33 is merely a coincidental infection, then both the current vaccines and the 9-valent vaccines could prevent this case of cervical cancer. While there would be an absolute benefit from the 9-valent vaccine, there would be no added benefit over the currently available vaccines. In contrast, if HPV type 33 is the only causative agent for cervical cancer and the presence of HPV type 18 is a coincidental infection, then the 9-valent vaccine could prevent this case, but the current vaccine would have no benefit (assuming no cross-protection against non-vaccine type).

If the cervical cancer in this case is caused by a synergistic effect between HPV-18 and -31, then both current and 9-valent vaccines could offer some protective benefit against the development of cervical cancer for this case, but the precise added benefit from the 9-valent vaccine over the current vaccine, if any, would be difficult to determine.

**Scenario Y**: A cervical cancer case with the presence of HPV types 18, 33, and 39

There is limited evidence to help determining what the causative agent(s) actually is(are) when there are multiple types of HPV present. In this case, it is possible that the 9-valent vaccine could offer a) no benefit at all (i.e., if HPV-39 is the sole causative agent for cervical cancer), b) absolute benefit but no incremental benefit compared to current vaccines (i.e., if HPV-18 is the only causative agent), c) both absolute benefit and incremental benefit (i.e., if HPV-33 is the only causative agent), or d) both absolute benefit and possible incremental benefit (i.e., HPV type 18, 33 and 39 all synergistically contribute to causing cervical cancer).

Scenarios X and Y demonstrate that the presence of multiple HPV infections in cervical cancer introduces complexity and uncertainties in estimating absolute and incremental benefits of the 9-valent vaccine. It is possible that for cancer cases with more than one oncogenic HPV type, the 9-valent vaccine could offer full preventive effect or, on the other extreme, could offer no preventive effect, depending on which type(s) is the causal agent, whether the 9-valent vaccine protects against the causal agent(s), and whether types interact synergistically to cause cancer.
